# Experimental pig-to-pig transmission dynamics for African swine fever virus, Georgia 2007/1 strain

**DOI:** 10.1017/S0950268815000862

**Published:** 2015-05-20

**Authors:** C. GUINAT, S. GUBBINS, T. VERGNE, J. L. GONZALES, L. DIXON, D. U. PFEIFFER

**Affiliations:** 1 Royal Veterinary College, Department of Production and Population Health, Hatfield, UK; 2 The Pirbright Institute, Pirbright, UK

**Keywords:** African swine fever, domestic pigs, basic reproduction number, modelling, transmission

## Abstract

African swine fever virus (ASFV) continues to cause outbreaks in domestic pigs and wild boar in Eastern European countries. To gain insights into its transmission dynamics, we estimated the pig-to-pig basic reproduction number (*R*_0_) for the Georgia 2007/1 ASFV strain using a stochastic susceptible-exposed-infectious-recovered (SEIR) model with parameters estimated from transmission experiments. Models showed that *R*_0_ is 2·8 [95% confidence interval (CI) 1·3–4·8] within a pen and 1·4 (95% CI 0·6–2·4) between pens. The results furthermore suggest that ASFV genome detection in oronasal samples is an effective diagnostic tool for early detection of infection. This study provides quantitative information on transmission parameters for ASFV in domestic pigs, which are required to more effectively assess the potential impact of strategies for the control of between-farm epidemic spread in European countries.

## INTRODUCTION

African swine fever virus (ASFV), a member of the Asfarviridae family, causing a severe haemorrhagic disease in domestic and wild swine is currently circulating in Eastern Europe [1, 2]. Controlling the spread remains challenging due to the lack of effective vaccines and treatments, the potentially long survival of the virus in the environment and the potential role of wild boar in virus spread [1]. African swine fever (ASF) also represents a serious concern for European pig-producing countries due to the cost of stamping-out policies and the economic burden that bans impose on international trade, especially as production and consumption of pork are expected to further increase in future [1, 3]. ASFV spreads by direct contact between infectious and susceptible animals [4–10]. Infection may also occur by indirect contact with, for example, contaminated surfaces of transport vehicles, contaminated clothing of animal workers or through feeding of swill that contains contaminated pig products [11–13]. Airborne transmission has been demonstrated over short distances [14]. For each of the potential routes for ASFV transmission between individual pigs, quantitative parameters have to be determined so that control programmes can be targeted based on the relative importance of each pathway.

The basic reproduction number (*R*_0_), defined as the average number of newly infected cases caused by one infectious individual during its infectious period in a susceptible population, is a key quantitative indicator of the transmission potential and therefore very useful for evaluating the likely impact of different components in control strategies [15–18]. So far, *R*_0_ for the currently circulating ASFV strain in Eastern Europe has been reported in only one study [19]. The authors developed a mathematical model to describe the transmission of ASFV within farms of the Russian Federation and estimated *R*_0_ at 9·8 [95% confidence interval (CI) 3·9–15·6] based on field and experimental data. However, estimates for transmission parameters strongly depend on assumptions regarding the length of the infectious, latent and incubation periods [20–23]. For example, different *R*_0_ estimates for classical swine fever virus (CSFV) were obtained depending on the diagnostic tool (detection of genome or infectious virus) or the biological marker (viraemia, infectious oropharyngeal or rectal fluids, clinical symptoms) used to determine the time of infection or the duration of infectiousness [24, 25].

The objective of the current study was to estimate quantitative pig-to-pig transmission parameters for the currently circulating ASFV strain in Eastern Europe. For this purpose, we fitted stochastic Susceptible-Exposed-Infectious-Removed (SEIR) models to detailed data available from transmission experiments, in which susceptible pigs were exposed by direct and indirect contact to pigs infected with the ASFV Georgia 2007/1 strain. Furthermore, we assessed the sensitivity of the estimates to assumptions about the time of infection and duration of infectiousness for the infected animals.

### Ethical standards

The authors assert that all procedures contributing to this work comply with the ethical standards of the relevant national and institutional guides on the care and use of laboratory animals. All experimental procedures, carried out under UK Home Office Licence number 70/7198, were approved by the Pirbright Ethical Review process. All animals were euthanized for animal welfare reasons.

## METHODS

### Experimental ASFV dataset

Data were obtained from experimental ASFV transmission in weaner pigs as described in Guinat *et al.* [26]. In short, four groups of pigs were allocated to different rooms (A, B, C, D) and randomly selected pigs were inoculated intramuscularly with the highly virulent Georgia 2007/1 ASFV strain (Table 1). The inoculated pigs were in direct contact with susceptible pigs within each pen in all rooms. Rooms B and C were each split into two adjacent pens allowing indirect contact between inoculated and susceptible pigs, most likely through airborne transmission and small amounts of urine and faeces passing under the fence. The partition between pens did not allow nose-to-nose contacts. Blood samples were collected every 2 days and oral and nasal fluid samples daily. Samples were tested for presence of live ASFV and ASFV genome by virus isolation and quantitative real-time polymerase chain reaction (qPCR), respectively. All inoculated animals became infected and contact transmission was observed in all experimental groups. The inoculated pigs were generally viraemic 1–3 days before starting to excrete ASFV genome through the nasal and oral routes. The within-pen and between-pen contact pigs generally started to excrete ASFV genome through nasal and oral fluid samples 1–4 days before showing viraemia. Table 2 summarizes the diagnostic results in blood samples during the study period for all rooms.

**Table 1.** Numbers of pigs used and transmission results with the Georgia 2007/1 African swine fever virus strain

	Room			
	A	B	C	D
Number of inoculated pigs	5	4	4	3
Number of within-pen contact pigs	5	4	4	3
Number of between-pen contact pigs	0	4	4	0
Number of newly infected pigs	5	8	8	3

**Table 2.** Results of African swine fever virus (ASFV) isolation in blood samples from transmission experiments with domestic pigs infected with the Georgia 2007/1 ASFV strain

Animals	Day post-inoculation																		
	0	1	2	3	4	5	6	7	8	9	10	11	12	13	14	15	16	17	18
Room A																			
WP contact	−			−		−		−		−		+	+*						
WP contact	−			−		−		−		−		−		+*					
WP contact	−			−		−		−		−		+		+*					
WP contact	−			−		−		−		−		+		+*					
WP contact	−			−		−		−		−		+		+*					
Inoculated	−			−		+		+		+*									
Inoculated	−			+		+		+		+*									
Inoculated	−			+		+		+*											
Inoculated	−			+		+	+*												
Inoculated	−			−		+		+	+*										
Room B																			
BP contact	−			−		−		−		−		+		+*					
BP contact	−			−		−		−		−		−		+		+*			
BP contact	−			−		−		−		−		+		+		+*			
BP contact	−			−		−		−		+		+*							
WP contact	−			−		−		−		+	+*								
WP contact	−			−		−		−		+	+*								
WP contact	−			−		−		−		+	+*								
WP contact	−			−		−		−		+	+*								
Inoculated	−			+		+	+*												
Inoculated	−			−		+		+	+*										
Inoculated	−			+		+	+*												
Inoculated	−			−		+	+*												
Room C																			
BP contact	−			−		−		−		−		−		−		−		−	+*
BP contact	−			−		−		−		−		−		−		−		+	+*
BP contact	−			−		−		−		−		−		+		+	+*		
BP contact	−			−		−		−		−		−		+		+*			
WP contact	−			−		−		−		+		+	+*						
WP contact	−			−		−		−		−		+	+*						
WP contact	−			−		−		−		+	+*								
WP contact	−			−		−		−		−		−		+	+*				
Inoculated	−			+		+		+	+*										
Inoculated	−			+		+		+*											
Inoculated	−			−		+		+		+		+	+*						
Inoculated	−			+		+	+*												
Room D																			
WP contact	−			−		−		−		+	+*								
WP contact	−			−		−		−		−		+		+*					
WP contact	−			−		−		−		−		+		+*					
Inoculated	−			+		+	+*												
Inoculated	−			+		+		+*											
Inoculated	−			+		+	+*												

WP, Within-pen contact pigs; BP, between-pen contact pigs; –, negative virus isolation in blood; +, positive virus isolation in blood.

* Day of euthanasia.

### Model assumptions

Three models were developed in this study, each assuming a different latent period (summarized in Table 3). In all models, the animals were assumed to be infectious as soon as ASFV was isolated from blood samples, supported by the fact that ASFV was mainly shed in blood [26]. Since data on the moment of infection for the contact pigs were lacking, they were considered infected before the onset of infectiousness using an assumed latent period (*L*, the time from infection to the onset of infectiousness [21]) of either 3 days (model 1), 4 days (model 2) or 5 days (model 3). This was based on values observed among the inoculated pigs (Table 2).

**Table 3.** Model inputs

Model inputs	Model 1	Model 2	Model 3
*β*_w_, within-pen transmission rate	Baseline value = 0·50 per day		
*β*_b_, between-pen transmission rate	Baseline value = 0·01 per day		
*L*, latent period duration*	3 days	4 days	5 days
Marker for inoculated pigs	Blood		
Marker for within-pen contact pigs			
Marker between-pen contact pigs			
*T*, infectious period duration	3–6 days		
	3–14 days		

* A 2-day latent period had also been considered (data not shown).

The infectious period (*T*) represents the time between the onset of infectiousness and death or recovery [21]. The transmission experiments did not allow *T* to be estimated because animals were euthanized for welfare reasons, resulting in censored values [± standard deviation (s.d.)] for *T* (e.g. average of 4·7 (±1·4), 2·4 (±0·7) and 3·0 (±1·2) days observed for the inoculated, within-pen and between-pen contact pigs, respectively [26]). Therefore, animals were considered infectious either for a time period of 3–6 days (minimum infectious period) usually reported in previous studies or for 3–14 days (maximum infectious period) which has occasionally been observed [27–29]. We assumed *T* was normally distributed, as is considered to be a biologically realistic distribution for the infectious period of a virus [30], either with a mean (±s.d.) of 4·5 (±0·75) days or 8·5 (±2·75) days.

The animals were assumed to mix randomly either within or between pens, i.e. to have equal opportunity for contact with each other [21], which was considered plausible given the limited room/pen size in the experiments [26]. The number of new infections per unit of time was assumed to be proportional to the product of the proportion (or frequency) of infectious animals and the number of susceptible animals [21] which was considered adequate to describe the transmission dynamics process [31, 32].

### Model analyses

**Fig. 1.**
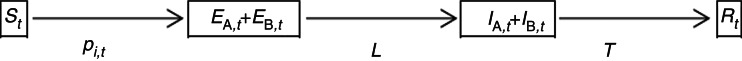
Schematic representation of the SEIR model used for estimating the experimental pig-to-pig transmission parameters for African swine fever virus. Considering two adjacent pens A and B at time *t*, pigs leave the susceptible compartment (*S*_*t*_) and become infected (*E*_A*,t*_ or *E*_B*,t*_) with probability *p*_*i,t*_. They leave the infected compartment and become infectious (*I*_A*,t*_ or *I*_B*,t*_) after *L* days. Finally, they leave the infectious compartment and die (*R*_*t*_) after *T* days.

We considered a standard transmission scenario with *I*_A*,t*_ and *I*_B*,t*_, the number of infectious pigs, *N*_A*,t*_ and *N*_B*,t*_ the total population size, in two pens A and B at time *t*, respectively. Pen A housed the inoculated pigs and the within-pen contact pigs, while pen B housed the between-pen contact pigs. We used a stochastic SEIR model to estimate the within- and between-pen transmission rate parameters (*β*_w_ and *β*_b_). In the model (Fig. 1), the population was divided into four classes over time *t* [21]: pigs leaving the susceptible compartment (*S*_*t*_) became infected (*E*_A*,t*_ and *E*_B*,t*_) with probability of infection *p*_*t*_, those leaving the infected compartment became infectious (*I*_A*,t*_ and *I*_B*,t*_) after *L* days, and those leaving the infectious compartment died (*R*_*t*_) after *T* days. The probability *p*_*t*_ depends on the number of infectious pigs (*I*_A*,t*_ and *I*_B*,t*_), the total population size (*N*_A*,t*_ and *N*_B*,t*_) and how frequently they make effective contact with each other within and between pens (*β*_w_ and *β*_b_), so that
1
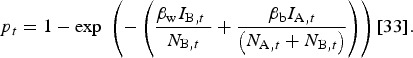

The number of new infections at time *t* (*C*_*t*_) follows a binomial distribution with the number of trials given by the susceptible population (*S*_*t*_) and the probability of success given by the probability of infection (*p*_*t*_). This was used to construct the likelihood for the data and we estimated *β*_w_ and *β*_b_ by maximizing this likelihood [34, 35]. In addition, 95% CIs were determined using ±1·96 times the standard error (s.e.) with the s.e. obtained from the inverse of the negative Hessian matrix for the log likelihood. Basic reproduction numbers (*R*_0w_ and *R*_0b_) were calculated using the relationship *R*_0w_ = *β*_w_*T* and *R*_0b_ = *β*_b_*T* [21] and the 95% CIs were based on the variance of *β*_w_, *β*_b_ and *T*. Models were implemented using R statistical software [35]. Models were compared based on Akaike's Information Criterion (AIC) [36].

### Vaccination coverage

Using the transmission parameter estimates, we calculated the vaccination coverage required to prevent pig-to-pig disease transmission based on the herd immunity threshold (HIT). HIT represents the minimum proportion of pigs that need to be immune in the population to limit ASFV transmission, given by HIT = 1 – (1/*R*_0w_) [21].

### Sensitivity analysis

We simulated ASF outbreaks in a pig unit to assess the effects of different assumptions regarding the different latent and infectious period durations. The weaner pigs on this farm unit are assumed to be housed in a ring of six adjacent pens, with 90 pigs in each (i.e. a total of 540 pigs). We simulated 1000 independent outbreaks, initiated by the introduction of one infected pig at time *t* = 0. For each simulation, *β*_w_, *β*_b_ and *T* were sampled from zero-truncated normal distributions under the assumption that these parameters are independently distributed. The mean and standard deviation for each distribution was defined so that 95% of the sampled values would fall within their respective 95% CI and *L* was considered to be either 3, 4 or 5 days. The likelihood surface for *β*_w_ and *β*_b_ suggested that the estimates for these parameters were not correlated (data not shown). We extended equation (1) to account for the fact that each pen is adjacent to two others, rather than one. Therefore, the probability that a susceptible pig in pen *j* becomes infected at time *t* is given by

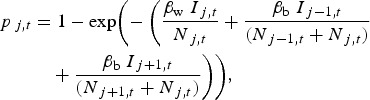

where *I*_*j,t,*_ *I_j_*_−1,*t*_ and *I*_*j*+1*,t*_ are the number of infectious animals in pens *j, j* – 1 and *j* + 1 at time *t*, respectively, and *N*_*j,t*_, *N_j_*_−1*,t*_ and *N*_*j*+1*,t*_ are the total number of pigs in these pens. From these simulations, we estimated the proportion of outbreaks that fail or result in limited numbers of infected animals, the number of newly infected pigs and the total number of infectious pigs per day. Simulations were implemented with R statistical software [35].

## RESULTS

### Transmission parameters for ASFV

Assuming blood as a biological marker for infectiousness, the increase in the latent period duration from model 1 to model 3 (i.e. from *L* = 3 to *L* = 5 days) resulted in an increase in *β*_w_ and *β*_b_ values (Table 4). The highest *β*_w_ and *β*_b_ estimates were obtained for model 3 with 0·9 (95% CI 0·4–1·3) and 0·4 (95% CI 0·2–0·7) newly infected pigs per day within and between pens, respectively. The lowest AIC was obtained for model 2, followed by model 3, and model 1 had the highest AIC. A similar pattern was observed for the *R*_0w_ and *R*_0b_ estimates rather than for the *β*_w_ and *β*_b_ estimates. In addition, the increase in the infectious period duration (i.e. from *T* = 3–6 to *T* = 3–14 days) also resulted in an increase in *R*_0w_ and *R*_0b_ values (Table 4). The highest *R*_0w_ and *R*_0b_ estimates were generated by model 3 showing that infectious pigs would cause up to 7·2 (95% CI 2·1–14·2) and 3·5 (95% CI 1·2–7·0) new infected cases within and between pens, respectively. *R*_0b_ estimates were generally lower than *R*_0w_ estimates, but not significantly greater than the threshold value of 1 in all models (except for model 3 with the maximum infectious period) considering the lower bound of the 95% CI. Similarly, the HITs reflected the *R*_0_ values, with the highest levels of vaccination required for model 3, i.e. from 74% (95% CI 47–84) to 86% (95% CI 55–93) depending on the infectious period duration.

**Table 4.** Maximum likelihood estimates (95% confidence intervals) for experimental pig-to-pig transmission parameters for Georgia 2007/1 African swine fever virus strain

Parameter	Model 1 (*L* = 3 days)	Model 2 (*L* = 4 days)	Model 3 (*L* = 5 days)
*β*_w_ (per day)	0·5 (0·3–0·7)	0·6 (0·3–1·0)	0·9 (0·4–1·3)
*β*_b_ (per day)	0·3 (0·1–0·4)	0·3 (0·1–0·5)	0·4 (0·2–0·7)
Minimum infectious period duration, *T* = 3–6 days			
*R*_0w_	2·2 (1·1–3·7)	2·8 (1·3–4·8)	3·9 (1·9–6·4)
*R*_0b_	1·2 (0·4–2·1)	1·4 (0·6–2·4)	1·9 (0·9–3·2)
HIT (%)	55 (9–73)	64 (23–79)	74 (47–84)
Maximum infectious period duration, *T* = 3–14 days			
*R*_0w_	4·0 (1·2–8·5)	5·3 (1·7–10·3)	7·2 (2·1–14·2)
*R*_0b_	2·0 (0·6–4·3)	2·5 (0·8–5·2)	3·5 (1·2–7·0)
HIT (%)	75 (17–88)	81 (41–90)	86 (55–93)
AIC	77·3	68·3	71·5

HIT, Herd immunity threshold; AIC, Akaike's Information Criterion.

### Simulation of ASFV outbreaks

The decrease in the latent period from model 3 to model 1 (i.e. from *L* = 5 to *L* = 3 days) resulted in an increase in the probability that an outbreak does not occur after ASFV is introduced into the farm and in the probability that this outbreak does not lead to the infection of all animals (Table 5). This effect was particularly marked for a shorter infectious period, i.e. *T* = 3–6 days. Decreasing the infectious period in all models from *T* = 3–14 days to *T* = 3–6 days resulted in an increase in the probability that the outbreak goes extinct (i.e. non-occurrence and incomplete population infection). This effect was particularly strong for a shorter latent period of *L* = 3 days (model 1). As a result, model 3 produced a 7% probability of outbreak failure and 10–17% probability of not all pigs on the farm becoming infected.

**Table 5.** Description of outbreaks simulated in a pig unit for Georgia 2007/1 ASFV strain

Parameter	Model 1 (*L* = 3 days)	Model 2 (*L* = 4 days)	Model 3 (*L* = 5days)
Minimum infectious period duration, *T* = 3–6 days			
Probability that outbreak does not occur after ASFV introduction to the farm	0·22	0·17	0·07
Probability that outbreak does not lead to infection of all population after ASFV introduction to the farm	0·65	0·45	0·17
Maximum infectious period duration, *T* = 3–14 days			
Probability that outbreak does not occur after introduction of ASFV to the farm	0·16	0·10	0·07
Probability that outbreak does not lead to infection of all population after ASFV introduction to the farm	0·27	0·18	0·10

ASFV, African swine fever virus.

**Fig. 2.**
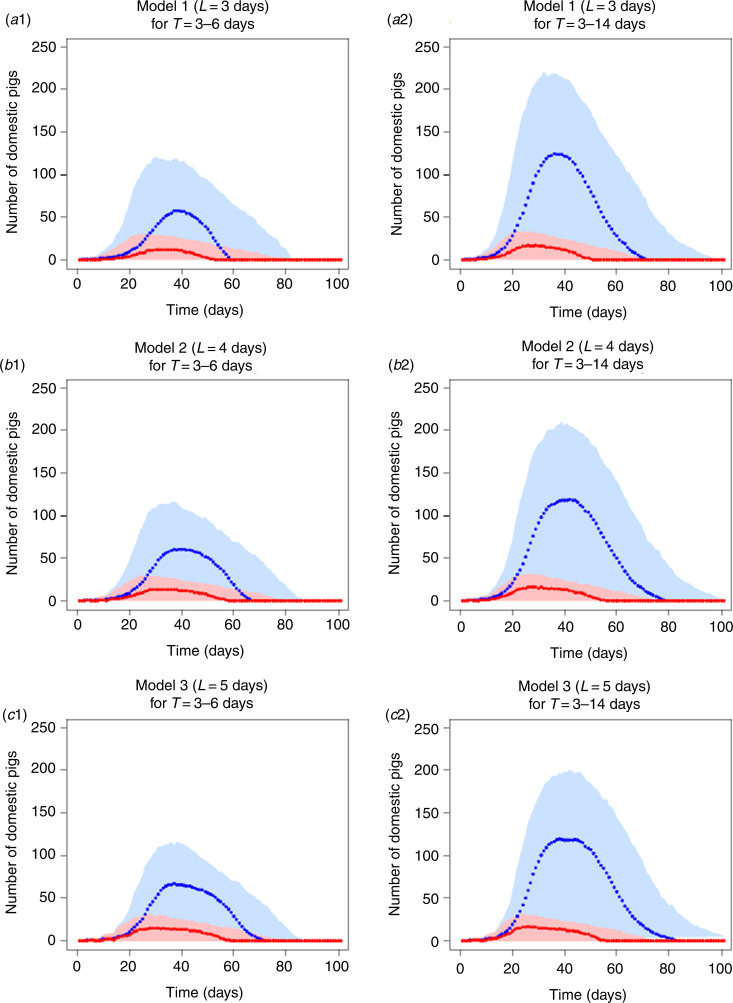
Median (dots) and 95% confidence intervals (shaded area) of the number of newly infected pigs (red) and of the total number of infectious pigs (blue) per day during simulated outbreaks within a farm unit with Georgia ASFV 2007/1 strain based on three different models. (*a*1, *a*2) Model 1 assumed a 3-day latent period. (*b*1, *b*2) Model 2 assumed a 4-day latent period. (*c*1, *c*2) Model 3 assumed a 5-day latent period. Infectious period duration (days) was represented as a normal distribution (mean ± standard deviation) of either 4·5 ± 0·75 days (*a*1, *b*1, *c*1) or 8·5 ± 2·75 days (*a*2, *b*2, *c*2).

Varying durations of latent and infectious periods resulted in similar epidemic curves (Fig. 2). For example, at the peak of the epidemic curve in all models, the number of newly infected pigs per day remained around 10 (95% CI 0–25) [Fig. 2(*a*1–*c*2)].

The increase in the latent period duration did not seem to modify the prevalence curve although it slightly increased the outbreak duration in all models, regardless of the infectious period duration (Fig. 2). For example, in model 3, the last infectious pigs were reported around 70 (95% CI 0–90) days compared to model 1 at around 60 (95% CI 0–80) days [Fig. 2(*c*1 *vs. a*1)]. The increase in the infectious period duration generally resulted in significantly higher numbers of infectious pigs per day and longer outbreak duration. For example, in model 2, more than 100 (95% CI 0–200) infectious pigs were observed at the peak of the prevalence curve and these animals were reported until 80 (95% CI 0–100) days when using the infectious period of 3–14 days (Fig. 2*b*2). When decreasing the infectious period to 3–6 days, approximately 50 (95% CI 0–100) infectious pigs remained at the peak of the prevalence curve and no infectious pigs were reported after 65 (95% CI 0–85) days (Fig. 2*b*1).

## DISCUSSION

This study provides quantitative estimates for the dynamics of pig-to-pig transmission for ASFV. Model 2, assuming a 4-day latent period and using presence of live virus in blood as a marker of infectiousness, had the smallest AIC value and was thus the model with the best fit to the data from the transmission experiments. For this model, the estimate for the transmission rate parameter (*β*) within a pen was 0·6 (95% CI 0·3–1·0) per day, while between pens it was 0·3 (95% CI 0·1–0·5) per day. The lower AIC for model 2 compared to models 1 and 3 suggests that 4 days might be a more appropriate latent period than 3–5 days. In the transmission experiments, this corresponds to the time when ASFV genome was first recovered in oro-nasal excretions from contact animals before they showed viraemia [26]. This means that the use of qPCR assay in oro-nasal samples from contact animals appears to be an effective tool for early detection of infection, as has previously been suggested for CSFV [25]. In addition, swabs are easier and cheaper to collect as well as requiring a less stressful sample collection procedure for the pigs than taking blood samples. However, it cannot be excluded that ASFV could be excreted through other routes or that contact pigs could be infectious after different days post-exposure that were not tested here. For example, in this study viraemia was considered closely related to efficiency of ASFV transmission, although the detection of live CSFV in oropharyngeal samples was reported to be a better indicator for infectiousness than blood samples [25]. This was not assessed in the present study due to the limited numbers of positive virus titration assay in oral samples.

Results demonstrate that, assuming a mean infectious period of 4·5 days, infectious pigs would infect on average 2·8 (95% CI 1·3–4·8) animals within their pen and 1·4 (95% CI 0·6–2·4) animals between pens. In the transmission studies, acute disease and fatal outcomes were observed for all individual infected pigs but the dynamics of the transmission process were relatively slow (Table 2), resulting in a prolonged epidemic at the herd level. In addition, specific or apparent clinical signs have been reported infrequently [26–28]. According to field observations, no particular clinical signs of illness or values of pig mortality were observed by animal keepers in most of the ASF cases [1]. Outcomes also showed that ASF outbreaks could go extinct within a pig unit, with 10–17% probability of outbreak failure and 18–45% probability of small-scale transmission. This therefore emphasizes the potential factors that make farmers or animal workers more likely to under-report suspicious ASF cases.

Estimates for the between-pen basic reproduction number (*R*_0b_) indicate that the disease spread among pigs is less efficient between than within pens. This suggests that adjustments to the design of the pen layout within a pig unit, such as better physically isolating neighbouring pens from each other, are likely to reduce pig-to-pig ASFV transmission. This probably also means that ASFV transmission occurs even more slowly between different pig houses or farms than was estimated among pigs in these transmission experiments. Although better pen separation should be feasible in pig farms [3], this will be more challenging in traditional free-ranging or backyard farms that are commonly found in Eastern Europe and where animals are not confined within a fenced area [1]. These findings also imply that animals moved from one pen to another should be assessed for health status under conditions of strict quarantine considering the latent period observed in animals. However, this will also be unrealistic in areas where lack of interest from stakeholders in eradicating the disease or poor compliance to biosecurity regulations are common [1]. In addition, design considerations for pig pens are unlikely to have practical impacts at the regional or national level for disease control strategies.

Our estimates for *R*_0_ are quite different from those reported in previous studies on ASFV transmission: *R*_0_ for the moderately virulent Malta strain was estimated to be 18·0 (95% CI 6·9–46·9) [37], while that for the highly virulent Russia strain was 9·8 (95% CI 3·9–15·6) [19]. This may reflect differences in virus strain, infection and infectiousness markers, pathogenesis, diagnostic tools, experimental conditions and estimation methods. Moreover, assumptions about when pigs became infected and how long they remained infectious influenced our estimates for the pig-to-pig transmission parameters and, hence, any conclusions about ASFV transmission within a farm (i.e. outbreak size and duration) and control (i.e. critical level of vaccination coverage). A study that focused on CSFV transmission found, for example, a significant difference between weaner and slaughter pigs, explained by potential various types of contact or susceptibility to the infection [34]. Although the clinical course of ASFV does not seem to be age dependent [28], infection transmission dynamics may also be influenced by host characteristics (e.g. sex or breed). Although the effect of housing systems on ASFV transmission remains unclear, the density and the contact structures that vary between backyard and different types of commercial pig farms are also likely to result in different pig-to-pig transmission parameters. This demonstrates the importance of considering the assumptions behind the model when the aim is to use the estimated values to draw inferences about the actual transmission dynamics [38, 39]. In the current study, intramuscularly inoculated pigs were used as virus sources for exposure of susceptible pigs. Although this route of inoculation has been shown to efficiently induce infection [40], it is not the natural route of transmission to susceptible pigs. This is likely to impact on our *R*_0_ estimates, particularly due to significant differences in the course of pathogenesis reported between intramuscularly inoculated pigs and pigs infected by contact [26]. As a result, secondary contact infection should be used in future experiments for generating *R*_0_ estimates that are a better representation of field conditions [41]. Therefore, more data from the field or from transmission experiments in relation to ASFV infection dynamics in domestic pigs are needed to be able to better understand the behaviour of the virus, especially in the context of different pig production systems.

This study provides insight into quantitative pig-to-pig transmission parameters for the currently circulating ASFV strain in Eastern European countries. The experimental conditions that were used are likely to match those observed in commercial weaner pig units with good sanitary measures and with the possibility of direct and indirect contact between pigs that are housed within several adjacent pens. Findings show a low to moderate transmissibility of ASFV between pigs and that the transmission is influenced by the contact structure between these animals. These estimated transmission parameters are essential to inform future mathematical models developed with the aim of predicting between-farm ASFV spread in Eastern European countries and beyond, which will also help policy-makers to optimize interventions that are available to impede the ASFV spread.
