# Experimental pig-to-pig transmission dynamics for African swine fever virus, Georgia 2007/1 strain– CORRIGENDUM

**DOI:** 10.1017/S0950268816001667

**Published:** 2016-12

**Authors:** C. GUINAT, S. GUBBINS, T. VERGNE, J. L. GONZALES, L. DIXON, D. U. PFEIFFER

The authors of the above mentioned article [1] were made aware after publication that the likelihood function defined in the R script used to implement the maximum likelihood contained an error.

The error has now been rectified in the original R script. As a result of this correction, the summary should read “Models showed that R_0_ is 5·0 [95% confidence intervals (CI): 2·4–9·1] within a pen and 2·7 (95% CI 0·7–5·2) between pens.” Tables 4 and 5 and Figure 2 have been revised and are presented below.

**Figure:**
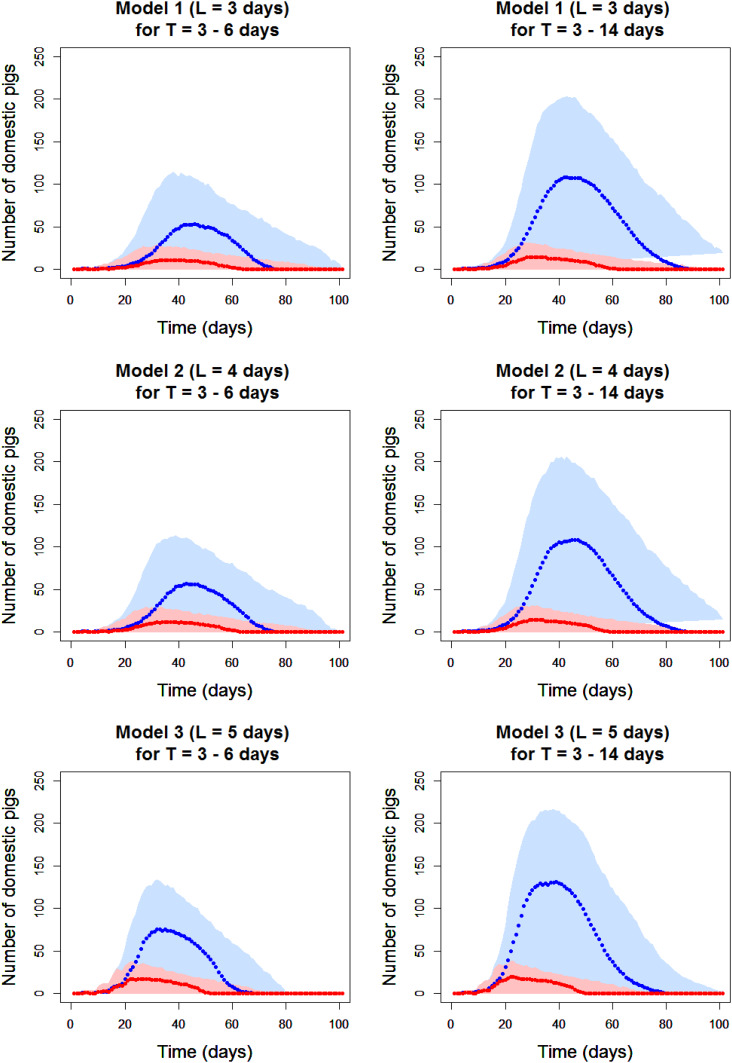

**Table 4.** Maximum likelihood estimates (95% confidence intervals) for experimental pig-to-pig transmission parameters for Georgia 2007/1 African swine fever virus strain

Parameter	Model 1 (L = 3 days)	Model 2 (L = 4 days)	Model 3 (L = 5 days)
β_w_ (per day)	0·60 (0·32–0·89)	0·62 (0·32–0·91)	1·17 (0·58–1·75)
β_b_ (per day)	0·36 (0·05–0·67)	0·38 (0·06–0·70)	0·61 (0·16–1·06)
Minimum infectious period duration, T = 3–6 days			
R_0w_	2·67 (1·43–4·56)	2·71 (1·32–4·56)	5·03 (2·38–9·12)
R_0b_	1·58 (0·13–3·16)	1·66 (0·28–3·31)	2·73 (0·70–5·19)
HIT (%)*	63 (30–78)	63 (19–78)	80 (58–89)
Maximum infectious period duration, T = 3–14 days			
R_0w_	4·87 (1·43–9·95)	4·99 (1·36–10·13)	9·28 (2·84–18·97)
R_0b_	2·80 (0·32–6·51)	3·07 (0·37–6·97)	4·83 (1·01–11·40)
HIT (%)*	79 (30–90)	80 (26–90)	89 (65–95)
AIC†	74·3	73·8	50·9

*Herd immunity threshold, †Akaike information criterion

**Table 5.** Description of outbreaks simulated in a pig unit for Georgia 2007/1 ASFV strain

Parameter	Model 1 (L = 3 days)	Model 2 (L = 4 days))	Model 3 (L = 5 days)
Infectious period duration, T = 3–6 days			
Probability that outbreak does not occur after introduction of ASFV in the farm	0·18	0·17	0·09
Probability that outbreak does not lead to infection of all population after introduction of ASFV in the farm	0·48	0·43	0·15
Infectious period duration, T = 3–14 days			
Probability that outbreak does not occur after introduction of ASFV in the farm	0·11	0·10	0·05
Probability that outbreak does not lead to infection of all population after introduction of ASFV in the farm	0·21	0·19	0·09

The discussion should read “Model 3, assuming a 5 day-latent period and using presence of live virus in blood as a marker of infectiousness, had the smallest AIC value and was thus the model with the best fit to the data from the transmission experiments.” and “Results demonstrate that, assuming a mean infectious period of 4·5 days, infectious pigs would infect on average 5·0 [95% confidence intervals (CI): 2·4–9·1] animals within their pen and 2·7 (95% CI 0·7–5·2) animals between pens.”

In addition, the authors would like to add information to clarify the methods which should help other researcher in re-producing the results:

First, equation 1 should read:

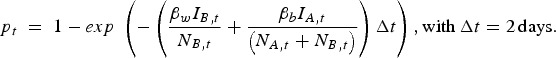


Second, an additional model assumption that was not clearly phrased in the original manuscript needs to be considered: animals were considered non-infectious on the day just prior to the day they were first tested positive (even though they were not tested on that day).

Finally, the authors provide with this corrigendum the data tables that were used to estimate the transmission parameters (see Tables S1, S2 and S3).

The authors would like to apologise for any inconvenience caused. They are very grateful to Lasse Engbo Christiansen, Josephine Perch Nielsen and Tinna Stokholm Larsen (DTU, Technical University of Denmark) for their careful review of our work and for bringing this error in the implementation of the methods to our attention.

## Supplementary material

For supplementary material accompanying this paper visit https://doi.org/10.1017/S0950268816001667. click here to view supplementary material
